# Genetic and environmental influences on quality of life: The COVID‐19 pandemic as a natural experiment

**DOI:** 10.1111/gbb.12796

**Published:** 2022-03-15

**Authors:** Margot P. van de Weijer, Dirk H. M. Pelt, Lianne P. de Vries, Floris Huider, Matthijs D. van der Zee, Quinta Helmer, Lannie Ligthart, Gonneke Willemsen, Dorret I. Boomsma, Eco de Geus, Meike Bartels

**Affiliations:** ^1^ Department of Biological Psychology, Vrije Universiteit Amsterdam Amsterdam The Netherlands; ^2^ Amsterdam Public Health Research Institute Amsterdam University Medical Centres Amsterdam The Netherlands

**Keywords:** covid‐19, heritability, natural experiment, quality of life, variance decomposition, well‐being

## Abstract

By treating the coronavirus disease 2019 (COVID‐19) pandemic as a natural experiment, we examine the influence of substantial environmental change (i.e., lockdown measures) on individual differences in quality of life (QoL) in the Netherlands. We compare QoL scores before the pandemic (*N* = 25,772) to QoL scores during the pandemic (*N* = 17,222) in a sample of twins and their family members. On a 10‐point scale, we find a significant decrease in mean QoL from 7.73 (SD = 1.06) before the pandemic to 7.02 (SD = 1.36) during the pandemic (Cohen's *d* = 0.49). Additionally, variance decomposition shows an increase in unique environmental variance during the pandemic (0.30–1.08), and a decrease in the heritability estimate from 30.9% to 15.5%. We hypothesize that the increased environmental variance is the result of lockdown measures not impacting everybody equally. Whether these effects persist over longer periods and how they impact health inequalities remain topics for future investigation.

## Graphical abstract

By treating the coronavirus disease 2019 (COVID‐19) pandemic as a natural experiment, we examine the influence of substantial environmental change (i.e., lockdown measures) on individual differences in quality of life (QoL) in the Netherlands. We compare QoL scores before the pandemic (*N* = 25,772) to QoL scores during the pandemic (*N* = 17,222) in a sample of twins and their family members. We find increased variance in QoL during the pandemic as the result of a large increase in unique environmental variance.

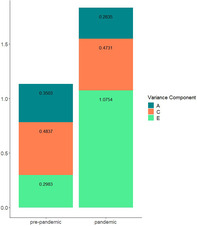

## 1 INTRODUCTION

Natural experiments pose a particularly interesting set of circumstances where an intervention is implemented that is not under the control of researchers.^1^ With respect to research in the domain of public health and human behaviour, a great advantage of research on natural experiments is that it corresponds to ‘real world’ conditions, in contrast to many controlled experiments. Additionally, natural experiment studies are essential for evaluating population‐scale (health) interventions and changes where experimental manipulation or random allocation is not feasible. As a result, natural experiments can provide unique ecologically valid insights into health processes as they are naturally occurring.

A well‐known example of a population‐level natural experiment is the compulsory schooling age reform in the United Kingdom, where the minimum age at which students were allowed to leave school increased from 15 to 16 for everyone born on or after 1 September 1957. An interesting finding in the context of this reform is that the additional year of education reduced the gap in unhealthy body size between those in the top and bottom terciles of genetic risk for obesity from 20 to 6 percentage points, thus benefitting those with a higher genetic risk for obesity.^2^ Another interesting set of natural experiments is the introduction of national tobacco control policies in different countries. For example, a workplace smoke‐free legislation was introduced in Ireland in March 2004. One of the results of this legislation was an immediate significant reduction in small‐for‐gestational birth rates, which was sustained over the post‐ban period.^3^ In the Netherlands, smoking prevalence decreased from 40%–51% to 22%–23% between 1993–1995 and 2009–2010, but no effect was seen on the heritability estimates of smoking.^4^

These examples involve national‐level policy changes aimed at improving population health. Another set of natural experiments is (natural) disasters with population‐level consequences. For example, on March 11, 2011, Japan was struck by an earthquake and consequent tsunami, leading to the loss of ±18,500 lives and ±345,000 people suffering damages to (or loss of) their house^5^ and many people suffered from posttraumatic stress disorder (PTSD) after this disaster. Hikichi and colleagues studied these events from a natural experiment perspective in order to gain knowledge on the association between social cohesion and the risk for PTSD.^5^ They found that individual‐ and community‐level social cohesion before the disaster were associated with a lower risk of showing PTSD symptoms following the disaster. Another disastrous event with population‐level consequences was World War 2 (WW2). During the horrific events of WW2, many children were separated from their parents. Pesonen and colleagues^6^ studied the effects of being separated from both parents or only one's father (because of military service) on depressive symptoms later in life (around 60 years of age) in a Finnish cohort. They found that being separated from both parents (but not from only the father) led to higher levels of depressive symptoms later in life, illustrating the prolonged effects of early life stress on later‐in‐life outcomes. These examples illustrate how natural experiments can provide novel insights that would have been difficult to study under ‘normal’ circumstances.

The difficulty in studying population‐level changes is that rapid, large‐scale policy or environmental changes are relatively rare. In the past year, large environmental changes occurred on a global scale because of the coronavirus disease 2019 (COVID‐19) pandemic. In March 2020, the World Health Organization (WHO) officially declared a pandemic, as the virus spread quickly across many countries in the world. As a result, many countries enforced a lockdown with varying levels of regulations. In the Netherlands, a so‐called ‘*intelligent lockdown*’ was installed, meaning that public spaces, schools, restaurants, and so forth were closed and that people were encouraged to work from home, but could still leave their house for walks and other outdoor activities. As a result, many people's lives changed profoundly from an economic, social, and physical perspective.

What these different aspects (economic, social, physical) have in common is that they are all related to mental health and well‐being. In a meta‐analysis by Prati and Mancini,^7^ the psychological impact of the COVID‐19 pandemic lockdowns across 25 studies was evaluated in terms of both positive and negative psychological functioning. They found that lockdowns had a small but detrimental effect on mental health, as expressed in negative psychological functioning (i.e., anxiety, depression, substance use, sleep disturbances, suicide risk, negative affect, and general distress), but surprisingly the effects on positive psychological functioning were not significant. In a Dutch sample, specifically people without severe or chronic mental health disorders showed a slight increase in depression, anxiety, worry, and loneliness symptoms, whereas people with depressive, anxiety, or obsessive–compulsive disorders did not seem to have increased symptom severity during the pandemic compared with before.^8^ Besides the effects of this large natural experiment on mean population levels of mental health, such an impactful natural experiment enables a unique study into causes of individual differences in mental health.

From a behaviour genetic perspective, the focus goes beyond mean level changes to explain the causes of individual differences. It is well established that individual differences in well‐being are influenced by both environmental factors and genetic factors: research indicates that about 40% of individual differences in well‐being is explained by genetic factors (the heritability), with the other 60% being explained by non‐shared/unique environmental factors.^9^ Research combining behaviour genetics and experiments is relatively scarce, and typically focuses on short‐term interventions. For example, one might use the ‘method of co‐twin control’, where only one member of an identical twin pair receives an intervention.^10^ This is an interesting way of studying the possible effect of the intervention while controlling for genetic confounding. Alternatively, we can study individual differences in the effect of an intervention by applying an intervention in a classical twin design (CTD). This design also provides information on stability and change of the sources of individual differences pre‐ and post‐intervention. For example, Haworth and colleagues examined the influence of a 10‐week positive psychology intervention on well‐being in a sample of 750 twins, and found that the relative influence of genetic and environmental influences remained stable, but that (partly) different non‐shared environmental factors influenced well‐being post‐intervention.^11^ In a more recent study, a brief online mindset intervention increased the relative influence of additive genetic factors to individual differences in mindset.^12^ The COVID‐19 pandemic can serve as a natural experiment for the investigation of absolute and relative changes in the genetic and environmental causes of variation in well‐being since we can compare the variance decomposition during the pandemic to before the pandemic. For two well‐being related constructs, optimism and meaning in life, it was already found that the heritability during the pandemic was slightly lower compared with before the pandemic.^13^ In addition to estimates of quantitative change such as lower heritability estimates, a study focusing on the qualitative aspects of the psychological responses to the COVID‐19 crisis in young adults found a genetic correlation of 1 between pre‐pandemic and pandemic purpose in life, indicating that the same genes affect this trait before and during the pandemic.^14^ Optimism and meaning in life can be viewed as facets of well‐being,^15^ but whether these effects are similar for other well‐being measures, such as quality of life (QoL), remains unexplored.

In the present study, we explore the impact of the COVID‐19 pandemic on individual differences in well‐being, quantified as QoL, in the Netherlands. We use a unique dataset that is comprised of data from twin families (e.g., twins, siblings, parents, aunts, uncles, nephews, nieces: pedigree data) both before and during the pandemic to provide a useful account of how genetic and environmental influences may be impacted by substantial environmental change.

## 2 MATERIALS AND METHODS

### 2.1 Participants

Participants were voluntary registrants of the Netherlands Twin Register (NTR).^16^ NTR participants are recruited through birth felicitation services, city councils, and online platforms. Every couple of years, biological and non‐biological family members are invited to partake in survey research on development, health, behaviour, and lifestyle. Relations among participants, that is, pedigree structure information, is stored in the ‘Person Administration of the Netherlands Twin Register’ (PANTER) database.^17^ Within this database, family roles and relations (e.g., mother–offspring, sibling–sibling) among participants are stored, with unlimited one‐to‐one relation possibilities for each individual. Participants can have multiple roles and relations in the database. For example, a person can be a mother and a twin.

For the current project, we selected a sample with pre‐pandemic QoL data, and a (partly overlapping) sample with pandemic QoL data. All participants were 16 years or older. For the pre‐pandemic sample, QoL data were available for multiple waves of data collection. If multiple observations were available for an individual, we selected the most recent pre‐pandemic observation (assessment data between January 2014 and February 2020). Within each family, if data for multiple siblings were available, we only selected data collected in the same data collection wave, in order to reduce potential time‐dependent confounders. Additionally, if data from both parent or spouses were available, we selected their data such that the data from both parents/spouses were included from the same wave of data collection.

During the pandemic, we made use of a single wave of data collection, which took place in April and May 2020, during the first lockdown in the Netherlands. Because we were interested in the effects of the lockdown on genetic and environmental influences on QoL, and not the effect of being infected itself, we excluded individuals with an (expected) COVID‐19 infection (see below for details). We included twins and higher‐order multiples (e.g., triplets), parents, siblings, and spouses (of multiples). Nuclear family information and age per type of family member is presented in Table 1. In total, pre‐pandemic QoL data were available for 25,772 individuals, and pandemic QoL data were available for 17,222 individuals, of whom 11,232 had data available at both time points. Across the whole sample, age ranged between 16 and 102. Figures S1 and S2 visualize the pre‐pandemic and pandemic age distributions, respectively.

**TABLE 1:** Pedigree composition

	Full data				Sample overlap two time‐points		
	Pre‐pandemic		Pandemic		Pre‐pandemic		Pandemic
	*N*	*M* (SD) age	*N*	*M* (SD) age	*N*	*M* (SD) age	*M* (SD) age
Families^a^	16,297	‐	11,960	‐	8317	‐	
Individuals	25,772	41.73 (14.57)	17222^b^	44.79 (14.70)	11,232	42.75 (15.30)	45.74 (14.66)
MZ males	1259	35.76 (17.56)	800	41.66 (17.71)	575	41.37 (18.69)	44.00 (17.41)
MZ females	3258	35.60 (16.28)	2522	38.93 (15.83)	1950	36.87 (16.84)	39.79 (15.67)
DZ males from DZ male pairs	896	30.09 (14.69)	481	35.94 (15.11)	329	33.82 (16.50)	37.04 (15.44)
DZ females from DZ female pairs	1740	30.99 (14.31)	1161	34.53 (13.82)	851	31.98 (14.90)	35.17 (13.66)
DZ males from DZ opposite‐sex twin pairs	855	29.82 (13.41)	480	35.53 (14.95)	323	33.50 (16.30)	36.76 (15.08)
DZ females from DZ opposite‐sex twin pairs	1488	27.95 (12.15)	940	32.01 (12.79)	689	29.17 (13.25)	32.58 (12.32)
Fathers	5709	49.87 (10.69)	2742	54.76 (10.97)	1762	53.36 (10.91)	56.35 (10.57)
Mothers	10,087	45.88 (10.07)	6677	48.58 (10.43)	4493	46.78 (10.29)	49.74 (10.21)
Brothers	148	37.99 (17.28)	291	42.99 (16.84)	71	42.56 (18.04)	45.13 (16.92)
Sisters	254	36.48 (17.06)	711	39.47 (15.41)	156	39.29 (17.91)	42.37 (16.68)
Spouses of twins	78	52.88 (13.10)	250	55.69 (12.40)	33	56.61 (12.77)	58.09 (12.47)

^a^ Families could exist of only one person.

^b^ There are some individuals that fall outside of the pre‐specified categories (i.e., children of twins), but these groups are very small.

### 2.2 Measures

#### 2.2.1 Quality of life

Well‐being was assessed as QoL using a Dutch version of Cantril's Self‐Anchoring Striving Scale.^18^ Participants were asked the question: ‘Where on the scale would you place your life in general? A score of 10 means the best life you can imagine, 1 means the worst life you can imagine’. In one of the pre‐pandemic questionnaires, the question was scored on a scale from 0 to 10 instead of 1 to 10. As almost no participant scored a 0 (*N* = 6) or 1 (*N* = 3) on this question, these two answers were pooled together as one so that the question was scored similarly from 1 to 10 across the different questionnaires.

#### 2.2.2 COVID‐19 infection status

COVID‐19 infection status was assessed by asking participants if they had been tested positive for COVID‐19 based on a PCR‐test. Additionally, since there was little testing in the Netherlands at the time of our pandemic data collection, we also enquired about the extent (on a 5‐point scale) to which participants had experienced a range of symptoms since February 20 and used the Menni self‐reported symptom‐based prediction model^19^, ^20^ to predict whether a person likely had COVID at the time of assessment. Detailed information on the development and application of this variable can be found in the original study paper.^20^ We excluded individuals from the pandemic sample if they reported having been tested positive (*N* = 85), or were predicted to have been infected based on the Menni model (*N* = 436).

### 2.3 Statistical analyses

#### 2.3.1 Pre‐pandemic to pandemic comparison

Means and standard deviations for pre‐pandemic and pandemic QoL for individuals with different roles within families were calculated using *R*.^21^ We selected a subsample of genetically unrelated individuals (*n* = 8529) and performed a paired‐samples *t* test to examine if QoL significantly changed from before to during the pandemic. Effect sizes were calculated using Cohen's *d* for paired samples. Additionally, we calculated within‐individual difference scores that reflect individual change from before to during the pandemic.

#### 2.3.2 Kinship correlations

Kinship correlations were obtained to acquire a first indication of familial resemblance for QoL during and before the pandemic. We calculated the kinship correlations using the Kinship Correlation Generator Tool (https://github.com/matthijsz/KinshipCorrelationGenerator). This tool uses a pedigree file with parent‐offspring relations and an individual level phenotype file as input to estimate correlations for different familial pairs, for example, mono‐ and dizygotic twins, parent‐offspring or cousin pairs. Weights are assigned to each pair of observations based on the number of times each individual is included in relation to different people. Per kinship relation we obtained: 1) a correlation between relatives for pre‐pandemic QoL, 2) a correlation for pandemic QoL, 3) a correlation between pre‐pandemic QoL in individual 1 and pandemic QoL in individual 2 for each pair of relatives, and 4) a correlation between pandemic QoL in individual 1 and pre‐pandemic QoL in individual 2 for each pair of relatives. Thus, correlation 1) and 2) were correlations within time‐points, while 3) and 4) were cross‐time correlations. These last two correlations between pre‐pandemic and pandemic QoL were pooled with fixed effect meta‐analysis in the *meta* package in *R* so that one cross‐phenotype correlation is computed to be used for further interpretation. As the tool does not provide standard errors or confidence intervals (CIs), these were calculated manually ($s_{r}=\sqrt{\frac{1-r^{2}}{n-2}}$).

#### 2.3.3 Genetic analyses

We used the Mendel 16.0 software package ‘Variance Components’ analysis option^22^ to decompose (co)variation in QoL into additive genetic (A), dominant genetic (D), common/household environmental (C), and unique environmental (E, also includes measurement error) sources of (co)variation. Effects of age and sex were regressed out prior to the Mendel analyses, and subsequent analyses were conducted on the residual QoL scores.^23^ Shared environmental influences were defined as influences that are shared by members of the same household. As we are examining adults only, most (adult‐aged) children within a nuclear family will not live in the same household. Therefore, a household effect was specified for spouses.

To perform variance decomposition in Mendel, three input files are required: 1. an input pedigree file, 2. a control file, and 3. a definition file.

1. The input pedigree file contains all the familial and phenotype data, grouped by family ID. Within the pedigree file, the following variables are required: family ID, person ID, and a Father and Mother ID, sex, and Twincode (an identifier for MZ twin pairs indicating which individuals are part of the same MZ twin pair). Genetic relationships between individuals within a pedigree with the same family ID are traced based on parental IDs (e.g., individuals within the same family with the same two parents are inferred to be full siblings). Our input pedigree file further specifies the household indicator field (in our case, spouse ID), and two fields for the (residualized) phenotype values for QoL before and during the pandemic.
2. The control file indicates all the analysis parameters. In our case, this includes the relevant variance components (A/C/D/E), the column names for the two quantitative traits present in the input pedigree file, the group factor specification (spousal household) and the way missing values are defined.
3. Lastly, the definition file provides information on non‐mandatory variables in the pedigree file: the variable types (factor/variable), and the associated levels and bounds. Mendel uses the control and definition files to read in the data from the input pedigree file, and estimates variance components based on variance–covariance matrices for relatives with different degrees of genetic relatedness based on classical biometrical genetics.^24^

We analysed four different models: 1) an ACDE model where C indicates the common household for spouses, 2) an ACE model, 3) an ADE model, and 4) an AE model. We compared the different nested models by comparing the log likelihood (LL) of the full ACDE model to the LL of the nested sub‐models using a −2 log likelihood (−2LL) test that approximately follows a *χ* ^2^ distribution. Genetic and environmental correlations between the variance components were calculated by dividing the covariance of between pre‐pandemic and pandemic QoL variables by the square root of its underlying variances.^25^ These genetic and environmental correlations reflect the extent to which similar genes and environmental factors influence QoL at the two time‐points.

## 3 RESULTS

### 3.1 Pandemic to pre‐pandemic comparison

Mean QoL scores for the different groups can be found in Table S1. Across the full sample, mean QoL decreased from 7.73 (SD = 1.06) before the pandemic to 7.02 (SD = 1.36) during the pandemic. A paired samples *t* test in an unrelated subsample (*n* = 8529) indicates this difference to be significant (*t*[8528] = 45.57, *p* < 2.2 × 10^−16^), indicating that QoL scores significantly decreased during the pandemic. The Cohen's *d* statistic (0.49) indicated a medium effect size. Individuals with pre‐pandemic data but without pandemic data (non‐responders) did not score differently on the pre‐pandemic QoL measure than individuals who provided data for both time‐points (responders) (*M* = 7.71, SD *=* 1.09).

**FIGURE 1:**
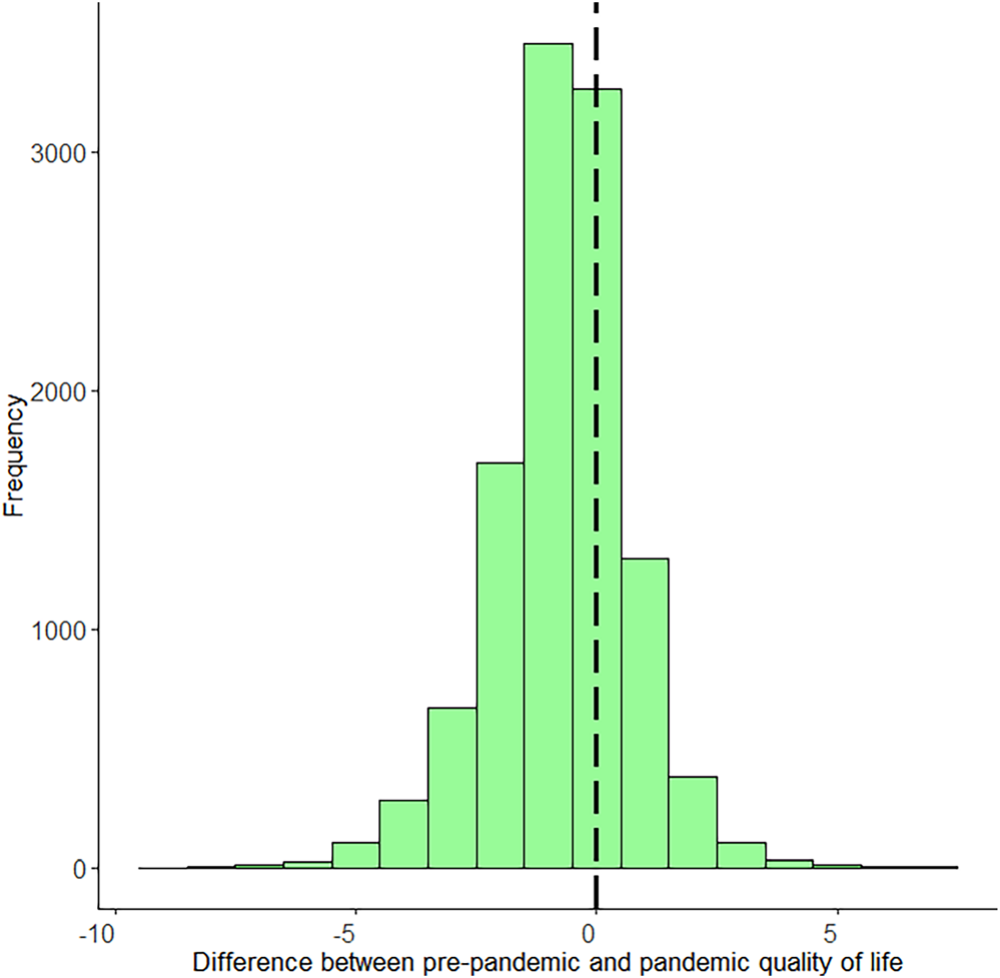
Histogram of quality of life (QoL) difference scores. The black dashed line indicates a change score of 0 or no change

Within‐individual change scores for the whole sample are visualized in Figure 1. A negative score indicates QoL decreased from before to during the pandemic, while a positive score indicates an increase in QoL. In total, QoL scores decreased for 6183 (55.05%) individuals, remained stable for 3239 (28.84%) individuals, and increased for 1810 (16.11%) individuals. From the group of individuals that indicated decreased QoL, 1158 (18.73%) individuals went from ‘sufficient’ QoL before the pandemic (indicated by a 6 or higher), to ‘insufficient’ QoL during the pandemic (indicated by a 5 or lower).

**FIGURE 2:**
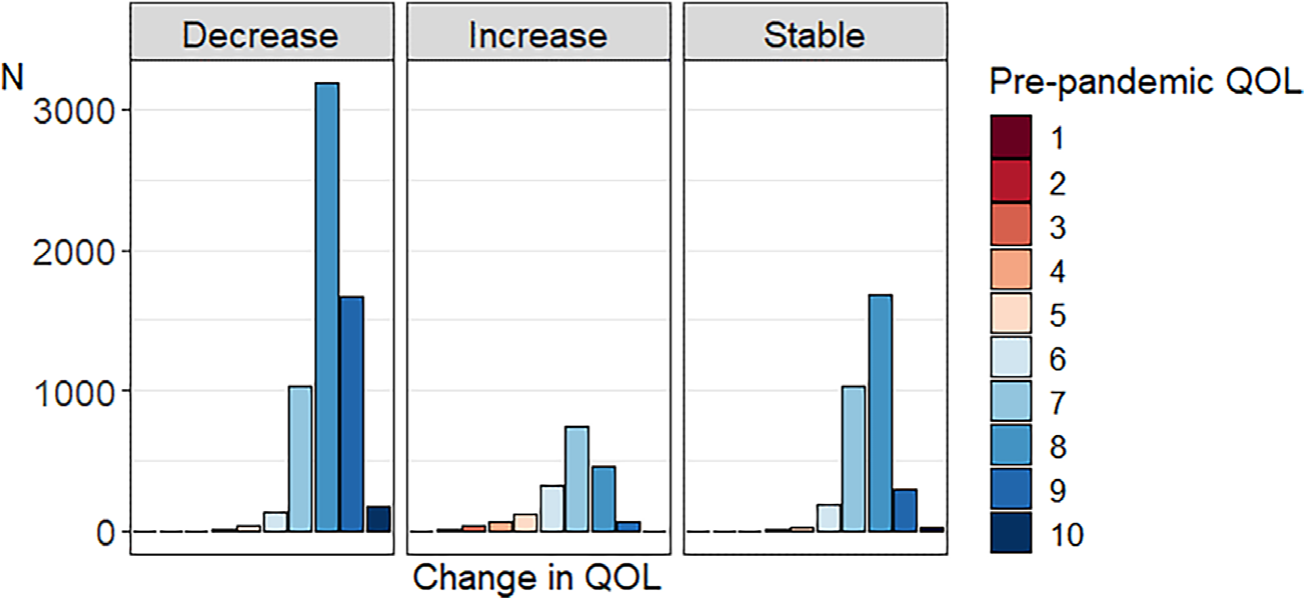
Number of individuals for whom quality of life (QoL) decreased, increased, and remained stable per pre‐pandemic QoL score

**FIGURE 3:**
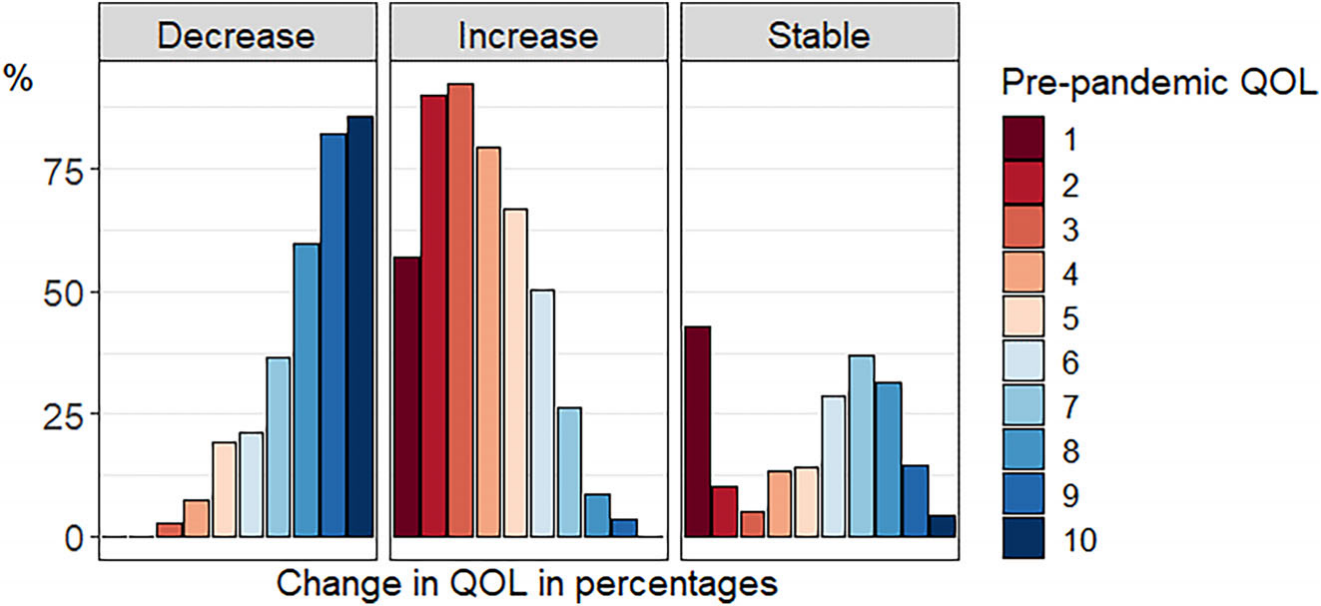
Percentage of individuals for whom quality of life (QoL) decreased, increased, and remained stable per pre‐pandemic QoL score. Each colour presents a pre‐pandemic QoL score, and is divided in percentages over the three change categories

Figures 2 and 3 depict the number of individuals and percentage of individuals, respectively, that increased, decreased, and remained stable per QoL pre‐pandemic score. As can be seen in Figure 2, pre‐pandemic QoL scores are relatively skewed with most people indicating good pre‐pandemic QoL. In general, the most common change was a decrease in QoL. Examining the group of respondents with decreased QoL during the pandemic in more detail (Figure 3), we see that individuals with high pre‐pandemic QoL scores more often decreased during the pandemic compared with individuals with lower pre‐pandemic QoL scores. With respect to the group of respondents that indicated increased QoL during the pandemic, it was especially individuals with lower pre‐pandemic QoL scores that indicated higher scores during the pandemic. We also plotted the percentage of individuals that decreased, increased, or remained stable for QoL for different age groups separately in Figure S3. Visual inspection of the plot does not show large differences between the age groups, with only a very slight trend of younger individuals being more negatively impacted in terms of QoL.

### 3.2 Longitudinal and kinship correlations

Across the whole sample, the correlation (*r*) between pre‐pandemic and pandemic QoL was 0.28 (*CI* = 0.26–0.30). Number of pairs for pre‐pandemic and pandemic QoL and correlations for the different relationship types are presented in Table 2. The pre‐pandemic MZ correlations for males (*r* = 0.46, *CI* = 0.37–0.55) were more than twice as high as the correlations for DZ male (DZM) pairs (*r* = 0.10, *CI* = −0.05–0.25), suggesting a role for additive and dominant genetic influences. Correlations for MZ females (*r* = 0.29, *CI* = 0.23–0.35) were slightly less than twice the female (DZF) pair correlations (*r* = 0.15, *CI* = 0.05–0.25), suggesting a role for additive genetic and shared environmental influences. The DZ opposite sex (DOS) pair correlation (*r* = 0.20, *CI* = 0.11–0.29) was slightly higher than the DZM and DZF correlations, albeit with overlapping CIs. The spousal correlation (*r* = 0.35, *CI* = 0.32–0.38) was relatively high, and was modelled as a common household variable in later analyses. Parent–offspring and sibling–sibling correlations were in the same range as DZ correlations.

**TABLE 2:** Kinship correlations for quality of life

	Pre‐pandemic			Pandemic			Cross‐correlation^a^	
	*N* pairs (weighted)	*r* (SE)	95% CI	*N* pairs (weighted)	*r* (SE)	CI	*r* (SE)	95% CI
Spouses (incl. parents of twins)	3951	0.35 (0.01)	0.32–0.38	1428	0.24 (0.03)	0.19–0.29	0.11 (0.02)	0.08–0.14
MZM	341	0.46 (0.05)	0.37–0.55	168	0.15 (0.08)	0–0.30	0.14 (0.05)	0.04–0.23
MZF	1063	0.29 (0.03)	0.23–0.35	719	0.21 (0.04)	0.14–0.28	0.11 (0.03)	0.06–0.16
DZM	178	0.10 (0.08)	−0.05–0.25	63	−0.03 (0.13)	−0.28–0.22	0.15 (0.08)	0–0.29
DZF	416	0.15 (0.05)	0.05–0.25	197	0.29 (0.07)	0.16–0.42	0.09 (0.05)	0–0.18
DOS	454	0.20 (0.05)	0.11–0.29	200	0.17 (0.07)	0.03–0.31	0.10 (0.04)	0.02–0.19
Mother–Daughter	1849	0.14 (0.02)	0.09–0.19	1209	0.03 (0.03)	−0.03–0.09	0.08 (0.02)	0.04–0.12
Mother–Son	1023	0.21 (0.03)	0.15–0.27	492	0.10 (0.04)	0.01–0.19	0.11 (0.03)	0.06–0.16
Father–Daughter	1174	0.15 (0.03)	0.09–0.21	736	0.04 (0.04)	−0.03–0.11	0.04 (0.02)	0–0.09
Father–Son	689	0.12 (0.04)	0.05–0.19	354	0.12 (0.05)	0.02–0.22	0.09 (0.03)	0.02–0.15
Brother–Brother	156	0.04 (0.08)	−0.12–0.20	71	0.05 (0.12)	−0.19–0.29	0.09 (0.08)	−0.07–0.24
Brother–Sister	650	0.18 (0.04)	0.10–26	444	0.08 (0.05)	−0.01–0.17	0.08 (0.03)	0.02–0.14
Sister–Sister	460	0.20 (0.05)	0.11–29	319	0.22 (0.05)	0.11–0.33	0.12 (0.04)	0.04–0.19

Abbreviations: CI, confidence interval; DOS, dizygotic opposite sex; DZF, dizygotic females; DZM, dizygotic males; MZF, monozygotic females; MZM, monozygotic males; *r*, correlation.

^a^ See Section 2 for an explanation of how the cross‐correlation was computed.

Pandemic QoL correlations were similar to or lower than pre‐pandemic QoL correlations. As seen in Table 2, twin‐ and spousal correlation estimates decreased, indicating a larger role for the non‐shared environment during the pandemic. An exception is the DZF correlation (*r* = 0.29, *CI* = 0.16–0.42), which seemed to increase. The overlapping CIs for most twin correlations do suggest that this might not be a significant decrease. A larger role for E was also suggested by the parent‐offspring correlations, with the correlations with daughters no longer being significantly different from zero. Sibling correlations were similar during and before the pandemic.

The separate cross‐time correlations (and sample sizes), and the meta‐analysed cross‐correlation estimates can be found in Tables S2 and 2. Correlations between pre‐pandemic and pandemic QoL were lower than the correlations for pre‐pandemic QoL, and comparable to pandemic QoL correlations. The relatively low correlations between pre‐pandemic and pandemic QoL suggest a large role for unique environmental influences, as these are not shared between different family members and thus introduce differences between family members.

### 3.3 Genetic analyses

The total phenotypic variance in QoL increased from 1.13 before the pandemic to 1.83 during the pandemic. The full model comparison results and variance decomposition for all the different models can be found in Tables S3 and S4. The best fitting model was the ACE model including additive genetic, common household for spouses, and unique environmental variance components. The variance component estimates for the ACE model can be found in Table 3. The increase in total variance is attributable to a large increase in unique environmental variance (from 0.30 to 1.08). While unique environmental variance increased, the common environmental variance remained stable, and the genetic variance decreased from 0.35 to 0.28.

**TABLE 3:** Unstandardized (incl. SE) and standardized variance components

	Pre‐pan	Pan	Pre‐pan	Pan	Pre‐pan	Pan	Pre‐pan	Pan
	A	A	C	C	E	E	P	P
*Unstandardized*								
Pre‐pan	0.3503 (0.0205)		0.4837 (0.0172)		0.2983 (0.0250)		1.1323	
Pan	0.1828 (0.0222)	0.2835 (0.0430)	0.1936 (0.0234)	0.4731 (0.0479)	0.0292 (0.0310)	1.0754 (0.0629)	0.4056	1.8320
*Standardized* (*unstandardized estimate/total phenotypic variance*)								
Pre‐pan	0.3094		0.4272		0.2634			
Pandemic	0.4507	0.1547	0.4774	0.2582	0.0720	0.5871		

Abbreviations: A, additive genetic variance; C, common environment variance; E, unique environment variance; P, phenotypic variance; pan, pandemic; pre‐pan, pre‐pandemic.

Consequently, the standardized variance decomposition of QoL also changed from before to during the pandemic. Relatively, the magnitude of the total variance that was explained by genetic differences, or the heritability, decreased from 30.9% to 15.5%, and the magnitude of common environmental influences decreased from 42.7% to 25.8%. Unique environmental factors became relatively more important, with 58.7% of the variance in QoL being explained by unique environmental factors during the pandemic, compared with 26.3% before the pandemic.

Most of the covariance between QoL before and during the pandemic is explained by genetic (45.1%) and shared environmental (47.7%) factors. Only 7.2% of the covariance was explained by unique environmental factors. Lastly, we found moderate genetic (*r*_A_ = 0.58) and common environmental correlations (*r*_C_ = 0.40), and a small unique environmental correlation (*r*_E_ = 0.05) between pre‐pandemic and pandemic QoL scores.

## 4 DISCUSSION

The present study set out to examine the impact of an impactful natural experiment (the COVID‐19 pandemic) on the genetic architecture of well‐being, measured as QoL. We find that on average, QoL decreased from 7.73 to 7.02 in the first months after the onset of the pandemic, reflecting a medium decrease (*d* = 0.49). QoL scores decreased for more than half the sample (55.05%), remained stable for 28.84% of the sample, and increased for 16.11% of the sample. Additionally, bivariate variance decomposition in Mendel showed a large increase in unique environmental variance during the pandemic. As a result, the relative proportion of individual differences explained by genetic factors (i.e., the heritability) decreased during the pandemic.

So far, the existing literature comparing pre‐pandemic and pandemic well‐being has produced mixed results. A meta‐analysis by Prati and Mancini^7^ did not find a significant effect on positive psychological functioning across six studies. However, positive psychological functioning was assessed in different countries using diverging well‐being definitions, for example, mental well‐being measured in the United Kingdom using the Short Warwick–Edinburgh Mental Wellbeing Scale,^26^ subjective well‐being was measured in France based on the frequency participants reported feeling ‘nervous’, ‘low’, ‘relaxed’, ‘sad’, ‘happy’, and ‘lonely’,^27^ and positive affect measured using the Positive Affect Negative Affect Scale in China.^28^ In a similar fashion, Aknin and colleagues^29^ performed a review on mental health during the pandemic and concluded that life satisfaction was largely unchanged in many countries during the first year of the pandemic, but that people did experience more unpleasant emotions during the pandemic.

In the present study, we find less optimistic results for QoL than expected based on these reviews, with the majority of individuals reporting lower QoL during the pandemic compared with before the pandemic. While the reason for this discrepancy is not clear, it might have something to do with the time period in which we collected the pandemic data in the Netherlands. Since the data were collected during the first lockdown accompanying the first wave of COVID‐19 infections, these results represent the first response of participants to the pandemic and consequent lockdown measures. In the earlier stages of the pandemic, there were many uncertainties and fears over the virus, infection rates were high, and the lockdown measures were highly disruptive. As such, changes in QoL may have been especially pronounced in these beginnings of the pandemic, and may have returned to more normal levels later on. It should be mentioned that some of the studies included in the reviews above also examined effects during the first lockdown. Therefore, it is likely that there are different effects across different countries, even during similar lockdown periods.

Important in the context of these results is that our sample, and the Netherlands in general, scores relatively high on QoL and other well‐being measures compared with other countries. The Netherlands scores among the top happiest countries according to the 2019 World Happiness Report,^30^ which was unchanged in the 2021 World Happiness Report that reported on the data collected in 2020 (during the pandemic).^31^ Importantly, we found a pandemic average QoL of 7.02 which is significantly lower than the pre‐pandemic average, but still a good score indicating that people were still quite satisfied with their QoL. We found that especially those with higher QoL scores were prone to decreases in QoL during the pandemic. Given the skewed distribution of QoL in our sample, this was the majority of our sample. Increases in QoL, however, were found mostly for individuals with lower QoL scores. While this is a relatively small part of our sample, it was surprising that it was especially individuals with lower baseline QoL that showed improvements in QoL during the pandemic. A potential explanation is that we only examined individuals that did not have a COVID‐19 infection around the time of assessment. Individuals with low levels of baseline QoL might have evaluated their QoL differently during the pandemic as they started comparing themselves with others that did become ill. In this way, they might have altered their perception, causing them to provide a different judgment during the pandemic.^27^ Individuals with higher levels of baseline QoL, on the other hand, might not have focused on these kinds of comparative mechanisms because they did not think their QoL was worse than average to begin with. Importantly, another possibility is that these findings might (partly) result from regression to the mean (RTM), the phenomenon whereby the second assessment of a trait results in values closer to the mean than at initial assessment purely by chance. However, pre‐pandemic QoL was measured on multiple occasions for some individuals, in which case we chose the latest available time‐point. By selecting participants from different measurement occasions, we attempt to acquire a better estimate of the participants' true baseline mean, which in turn decreases RTM.^32^ In a way, our pre‐pandemic QoL measure is formed by taking a random sample around an individual's baseline mean levels. This makes it less likely that high/low scorers will inevitably go down/up at the next measurement occasion (i.e., during the pandemic).Therefore, while we cannot rule out regression to the mean completely, this does make it less likely that our results are (fully) attributable to this phenomenon.

We used Mendel, instead of the CTD, to decompose the variance into genetic and environmental sources of variation. In the CTD, the variance components are estimated based only on the MZ and DZ twin covariances. As a result, only three parameters can be estimated simultaneously, so that an a priori choice needs to be made between an ACE or ADE model. The advantage of the Mendel software is that it allows for efficient analysis of whole pedigree data, allowing us to examine a large sample and estimating A, C, D, and E simultaneously. There are extensions of twin designs where other family members can be included, such as the Cascade model^33^ that are more flexible in terms of model specification (e.g., constraining paths and sex‐specific heritability). However, the advantage of Mendel is that it easily allows for the inclusion of complex family relations and irregular pedigrees, as are present in large twin‐family registers like the Netherlands Twin Register. Yet, we did not find any evidence for dominant genetic effects (D), that is, alleles acting in a multiplicative fashion (dominance or epistasis). Based on the correlations between the different types of family members (Table 2), there was some suggestive evidence for D in the male twin correlations, but not the female twin correlations (which were based on a much larger sample). Results from existing twin‐ and family studies on the contribution of non‐additive genetic effects to well‐being have been very mixed, but the largest twin‐family study to date did find evidence for non‐additive genetic effects.^34^ Importantly, the study by Nes and colleagues focused on a happiness measure, while in the current study we examined individual differences in QoL, potentially explaining this discrepancy. This is in line with our earlier work where we also report stronger evidence for dominant genetic effects on happiness versus quality of line.^35^

A striking finding was the large increase in unique environmental variance—estimates of E more than doubled—during the pandemic, which resulted in a decreased heritability, indicating an increase of the relative importance of unique environmental factors. Similar results were found in a large etiological study by Carroll and colleagues showing that unique environmental influences were amplified for emotional symptoms and conduct problems in youth (but not for attention‐deficit hyperactivity problems) as a result of pandemic‐relation disruption among multiple life domains.^36^ This phenomenon, where the total genetic/environmental variance is dependent on the environment, in this case pandemic‐related environmental change, is reflective of quantitative gene–environment interaction (GxE). Our quantitative GxE finding is in line with the bioecological model that postulates that genetic influences are maximized in stable and adaptive environments, and non‐shared environmental influences are greatest in more ‘risky’ environments.^37^ Clearly, pandemics such as the COVID‐19 pandemic, can be viewed as more risky environments characterized by high levels of disruption and uncertainty about the future. Alternatively, the findings can also be framed in a social control model, where genetic influences are relatively dampened as the result of social constraints imposed by the environment.^37^ While it was clear at the beginning of this study that the lockdown introduced many social constraints and consequently large environmental change in people's everyday lives, we did not yet know whether this would lead to an increase or a decrease in environmental variance. Theoretically, the restrictions imposed by the lockdown measures could have reduced the environmental variance by making everyone's lives more similar to each other. However, the finding that these measures led to a large environmental increase suggests that the pandemic and consequent lockdown measures did not impact everybody in a similar way. It is important to identify such factors, since they potentially enlarge health inequalities during the pandemic. For example, a study by Ravens‐Sieberer found that children with low socioeconomic status, migration background, and limited living space were affected significantly more by the pandemic in terms of health‐related QoL, mental health problems, anxiety, and depression.^38^

Several potential explanations for individuals' different reactions to the environmental change imposed by the pandemic can be proposed. For example, people were encouraged to work from home, but only if possible. Before the pandemic, 1 in 3 people in the Netherlands (occasionally) worked from home. This increased to 1 in 2 people in the beginning of the pandemic, with the strongest increase in people with higher educational attainment and people using public transport to commute between home and work.^39^ Thus, the ‘working from home’ policy did not affect everyone in the population equally, potentially leading to increased differences in reported QoL across individuals. Additionally, with schools and day‐care centres closed, people with children were likely impacted in a different way than people without children. Parents working from home (with children also staying at home) presumably had more trouble concentrating and were less productive, but the extent to which depended on different factors, like the age of the child and the age of parents.^40^ While the present study cannot pinpoint what exactly caused the increased environmental variance, these factors might serve as suggested causes of increased environmental variance in QoL in follow‐up research.

Based on the variance and covariance estimates provided by Mendel, we were able to calculate genetic and environmental correlations, which tells us something about the extent to which the same genetic and environmental factors influence QoL at the two time‐points. We found a moderate genetic correlation (*r*_A_ = 0.58) and common environmental correlation (*r*_C_ = 0.40), and a small unique environmental correlation (*r*_E_ = 0.05). To test if a correlation is significantly different from zero (or one), one would normally fit a model where the relevant correlation is constrained to zero (or one) and compare the fit of this model to the fit of the unconstrained model. Unfortunately, as Mendel does not allow for the inclusion of such constraints, we were not able to perform such model comparisons. However, based on the point estimate (*E*_cov_ = 0.029) and standard error (SE = 0.031) of the unique environmental covariance, we can conclude that the unique environmental correlation is not significantly different from zero. In other words, the unique environmental factors influencing individual differences in QoL during the pandemic are completely different from the unique environmental factors influencing individual differences in QoL before the pandemic. This is important to consider when thinking about potential (positive) psychological prevention and intervention strategies to harness people from the negative effects of extreme environmental change, such as the COVID‐19 pandemic lockdowns. These strategies rely on existing research by focusing their strategies on existing evidence on correlates of well‐being. However, as indicated by this study, the environmental factors that determine individual differences in well‐being under ‘normal circumstances’ are likely not the same as during crisis situations like these.

It is important to interpret these results within the context of our sample and the time‐frame in which we collected the data. Since it was the first lockdown, when the WHO had just announced a pandemic, individuals were likely still psychologically adjusting to the new situation. Whether the effects found in this study would be similar in later stages of the pandemic is a question that remains to be answered. Additionally, different countries employed different strategies to contain the virus, with the Netherlands installing the intelligent lockdown where people were encouraged to stay at home, but were still allowed to freely move around outside at all times of day. In this light, the finding of the large increase in environmental variance is even more remarkable, as the regulations in the Netherlands were less strict than those in many other countries. As such, the environmental effects of more stringent lockdown measures may be even larger. In any case, it is reasonable to expect that countries with different regulations will find different results than presented here, as these regulations impact the extent to which people had to alter their lives. Finally, a limitation of our sample was that we had more female respondents than male respondents in both the pre‐pandemic sample (65% female, 35% male), and the pandemic sample (71% female, 29% male). The representativeness was further limited by there being roughly twice as many highly educated individuals in the sample than expected based on the Dutch population.

## 5 CONCLUSIONS

In conclusion, in this study we used data from before and during the COVID‐19 pandemic as a natural experiment to add to our understanding of genetic and environmental influences on QoL. By treating the COVID‐19 pandemic as a natural experiment, we were able to show the dynamics of environmental change on individual differences and heritability. The most prominent finding to emerge is that unique environmental factors became relatively more important in explaining differences in QoL during the pandemic, with genetic factors becoming less important. Additionally, it seems that different unique environmental factors become relevant to QoL during compared with before the pandemic. Further research is required to determine if these effects are similar in the long term. Additionally, future research should explore what environmental factors are important for QoL during the pandemic, as these factors likely increase health inequalities in the population.

## CONFLICT OF INTEREST

The authors declare no relevant financial or non‐financial interests to disclose.

## ETHICS APPROVAL

All procedures performed in studies involving human participants were in accordance with the ethical standards of the institutional and/or national research committee and with the 1964 Helsinki declaration and its later amendments or comparable ethical standards. This article does not contain any studies with animals performed by any of the authors. Ethical approval was provided by 1) the Ethical Review Board (VCWE) of the Faculty of Behavior and Movement Sciences of the VU University Medical Centre Amsterdam (VCWE‐2020‐083R1) and, 2) The European Research Council Executive Agency Screening Ethics Panel.

## CONSENT TO PARTICIPATE

Written informed consent was obtained from all individual participants included in the study.

## Supporting information


**FIGURE S1** Histogram of the pre‐pandemic sample age distribution.

**FIGURE S2** Histogram of the pandemic sample age distribution.

**FIGURE S3** Change in Quality of Life from before to during the pandemic per age group, in percentages.

**TABLE S1** Mean quality of life scores for the different groups.
**TABLE S2** Cross‐correlations used for cross‐correlation meta‐analysis.
**TABLE S3** Model comparisons of the different variance decomposition models.
**TABLE S4** Unstandardized variance decomposition of the different models.

## References

[1] Leatherdale ST . Natural experiment methodology for research: a review of how different methods can support real‐world research. Int J Soc Res Methodol. 2019;22:19‐35.

[2] Barcellos SH , Carvalho LS , Turley P . Education can reduce health differences related to genetic risk of obesity. Proc Natl Acad Sci USA. 2018;115:E9765‐E9772. 30279179 10.1073/pnas.1802909115 PMC6196527

[3] Kabir Z , Daly S , Clarke V , Keogan S , Clancy L . Smoking ban and small‐for‐gestational age births in Ireland. PLoS One. 2013;8:e57441. 23555561 10.1371/journal.pone.0057441 PMC3608631

[4] Vink JM , Boomsma DI . Interplay between heritability of smoking and environmental conditions? A comparison of two birth cohorts. BMC Public Health. 2011;111(11):1‐7. 10.1186/1471-2458-11-316 PMC3112130 21569578

[5] Hikichi H , Aida J , Tsuboya T , Kondo K , Kawachi I . Can community social cohesion prevent posttraumatic stress disorder in the aftermath of a disaster? A natural experiment from the 2011 Tohoku earthquake and tsunami. Am J Epidemiol. 2016;183:902‐910. 27026337 10.1093/aje/kwv335 PMC4867157

[6] Pesonen A‐K , Räikkönen K , Heinonen K , Kajantie E , Forsén T , Eriksson JG . Depressive symptoms in adults separated from their parents as children: a natural experiment during world war II. Am J Epidemiol. 2007;166:1126‐1133. 17875582 10.1093/aje/kwm254

[7] Prati G , Mancini AD . The psychological impact of COVID‐19 pandemic lockdowns: a review and meta‐analysis of longitudinal studies and natural experiments. Psychol Med. 2021;51:201‐211. 33436130 10.1017/S0033291721000015 PMC7844215

[8] Pan KY , Kok AAL , Eikelenboom M , et al. The mental health impact of the COVID‐19 pandemic on people with and without depressive, anxiety, or obsessive‐compulsive disorders: a longitudinal study of three Dutch case‐control cohorts. Lancet Psychiatry. 2021;8:121‐129. 33306975 10.1016/S2215-0366(20)30491-0 PMC7831806

[9] van de Weijer M , de Vries L , Bartels, M . Happiness and wellbeing; the value and findings from genetic studies. *Psyarxiv* ; 2021.

[10] Plomin R , Haworth CMA . Genetics and intervention research. Perspect Psychol Sci. 2010;5:557‐563. 25419226 10.1177/1745691610383513 PMC4239660

[11] Haworth CMA , Nelson SK , Layous K , et al. Stability and change in genetic and environmental influences on well‐being in response to an intervention. PLoS One. 2016;11:e0155538. 27227410 10.1371/journal.pone.0155538 PMC4881940

[12] Burgoyne AP , Carroll S , Clark DA , et al. Can a brief intervention alter genetic and environmental influences on psychological traits? An experimental behavioral genetics approach. Learn Motiv. 2020;72:101683.

[13] de Vries L , van de Weijer M , Pelt D , et al. Individual differences in the effect of the COVID‐19 pandemic on optimism and meaning in life. *Psyarxiv* ; 2021.

[14] Rimfeld K , Malanchini M , Allegrini AG , et al. Genetic correlates of psychological responses to the COVID‐19 crisis in young adult twins in Great Britain. Behav Genet. 2021;51:110‐124. 33624124 10.1007/s10519-021-10050-2 PMC7902241

[15] Kafka GJ , Kozma A . The construct validity of Ryff's scales of psychological well‐being (SPWB) and their relationship to measures of subjective well‐being. Soc Indic Res. 2002;57:171‐190.

[16] Ligthart L , van Beijsterveldt CEM , Kevenaar ST , et al. The Netherlands twin register: longitudinal research based on twin and twin‐family designs. Twin Res Hum Genet. 2019;22(6):623‐636. 31666148 10.1017/thg.2019.93

[17] Boomsma DI , Willemsen G , Vink JM , et al. Design and implementation of a twin‐family database for behavior genetics and genomics studies. Twin Res Hum Genet. 2008;11:342‐348. 18498212 10.1375/twin.11.3.342

[18] Cantril H . The Pattern of Human Concerns. Rutgers University Press; 1965.

[19] van Blokland IV , Lanting P , Ori APS , et al. Using symptom‐based case predictions to identify host genetic factors that contribute to COVID‐19 susceptibility. PLoS One. 2021;16:e0255402. 34379666 10.1371/journal.pone.0255402 PMC8357137

[20] Menni C , Valdes AM , Freidin MB , et al. Real‐time tracking of self‐reported symptoms to predict potential COVID‐19. Nat Med. 2020;26:1037‐1040. 32393804 10.1038/s41591-020-0916-2 PMC7751267

[21] RStudio Team . RStudio: Integrated Development Environment for R; Boston, MA: RStudio Team; 2021. http://www.rstudio.com/

[22] Lange K , Papp JC , Sinsheimer JS , Sripracha R , Zhou H , Sobel EM . Mendel: the Swiss army knife of genetic analysis programs. Bioinformatics. 2013;29:1568‐1570. 23610370 10.1093/bioinformatics/btt187 PMC3673222

[23] Baselmans BML , Willems YE , van Beijsterveldt CEM , et al. Unraveling the genetic and environmental relationship between well‐being and depressive symptoms throughout the lifespan. Front Psychiatry. 2018;9:261. 29962975 10.3389/fpsyt.2018.00261 PMC6010548

[24] Lange K , Boehnke M . Extensions to pedigree analysis. IV. Covariance components models for multivariate traits. Am J Med Genet. 1983;14:513‐524. 6859102 10.1002/ajmg.1320140315

[25] Posthuma D , Beem AL , de Geus EJC , et al. Theory and practice in quantitative genetics. Twin Res. 2003;6:361‐376. 14624720 10.1375/136905203770326367

[26] Kwong A , Pearson R , Adams M , et al. Mental health during the COVID‐19 pandemic in two longitudinal UK population cohorts. *medRxiv* 2020.06.16.20133116; 2020. 10.1192/bjp.2020.242 PMC7844173 33228822

[27] Recchi E , Ferragina E , Helmeid E , et al. The “eye of the hurricane” paradox: an unexpected and unequal rise of well‐being during the Covid‐19 lockdown in France. Res Soc Stratif Mobil. 2020;68:100508. 32834344 10.1016/j.rssm.2020.100508 PMC7247999

[28] Li HY , Cao H , Leung DYP , Mak YW . The psychological impacts of a covid‐19 outbreak on college students in China: a longitudinal study. Int J Environ Res Public Health. 2020;17(11):3933. 10.3390/ijerph17113933 PMC7312488 32498267

[29] Aknin L , de Neve J‐E , Dunn E , et al. Mental health during the first year of the COVID‐19 pandemic: a review and recommendations for moving forward. *PsyArXiv* ; 2021 10.1177/17456916211029964 PMC9274782 35044275

[30] Helliwell JF , Huang H , Wang S . Changing world happiness. In: Helliwell JF , Layard R , Sachs J , eds. World Happiness Report. Vol 2019; New York: Sustainable Development Solutions Network; 2019:11‐46.

[31] Helliwell JF , Huang H , Wang S , Norton M . Happiness, trust, and deaths under COVID‐19. In: Helliwell JF , Layard R , Sachs J , De Neve J‐E , eds. The World Happiness Report 2021; New York, NY: Sustainable Development Solutions Network; 2021.

[32] Barnett AG , van der Pols JC , Dobson AJ . Regression to the mean: what it is and how to deal with it. Int J Epidemiol. 2005;34:215‐220. 15333621 10.1093/ije/dyh299

[33] Keller MC , Medland SE , Duncan LE , et al. Modeling extended twin family data I: description of the cascade model. Twin Res Hum Genet. 2009;12:8‐18. 19210175 10.1375/twin.12.1.8 PMC4070287

[34] Nes RB , Czajkowski N , Tambs K. Family matters: Happiness in nuclear families and twins. Behavior Genetics. 2010;40(5):577–590. 10.1007/s10519-010-9365-x 20440640

[35] Bartels M , Boomsma DI . Born to be happy? The etiology of subjective well‐being. Behav Genet. 2009;39:605‐615. 19728071 10.1007/s10519-009-9294-8 PMC2780680

[36] Carroll SL , Shewark EA , Hyde LW , Klump KL , Burt SA . Understanding the effects of the COVID‐19 pandemic on youth psychopathology: genotype–environment interplay. Biol Psychiatry Glob Open Sci. 2021;1:345‐353. 34514460 10.1016/j.bpsgos.2021.07.004 PMC8415869

[37] South SC , Hamdi NR , Krueger RF . Biometric modeling of gene‐environment interplay: the intersection of theory and method and applications for social inequality. J Pers. 2017;85:22‐37. 26426103 10.1111/jopy.12231 PMC4818206

[38] Ravens‐Sieberer U , Kaman A , Erhart M , Devine J , Schlack R , Otto C . Impact of the COVID‐19 pandemic on quality of life and mental health in children and adolescents in Germany. Eur Child Adolesc Psychiatry. 2021;1:1‐11. 10.1007/s00787-021-01726-5 PMC7829493 33492480

[39] Hamersma M , de Haas M , Faber R . Thuiswerken en de coronacrisis: Een overzicht van studies naar de omvang, beleving en toekomstverwachting van thuiswerken in coronatijd. Ministerie van Infrastructuur en Waterstaat; 2020.

[40] Kesselring M , van Spanje‐Hennes A . Slepend en soms ook slopend. De impact van (de maatregelen tegen) COVID‐19 op het gezinsleven. Hogeschool Utrecht; 2021.

