# Supplementary figures and images for: Genetic and environmental influences on quality of life: The COVID‐19 pandemic as a natural experiment
Source: Genes Brain Behav. 2022 Mar 15;21(8):e12796. doi: 10.1111/gbb.12796 (PMC9111595; doi:10.1111/gbb.12796)

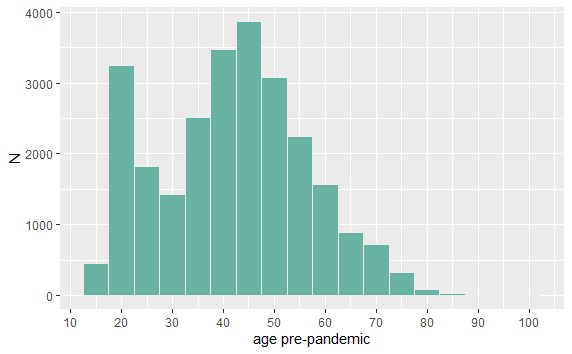

Supplement: Supplementary file 1 — FIGURE S1 Histogram of the pre‐pandemic sample age distribution. [file GBB-21-e12796-s002.png]

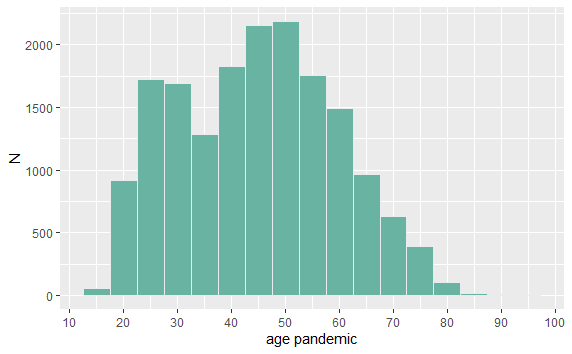

Supplement: Supplementary file 2 — FIGURE S2 Histogram of the pandemic sample age distribution. [file GBB-21-e12796-s004.png]

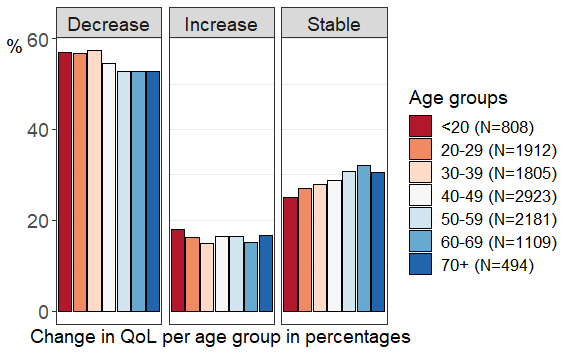

Supplement: Supplementary file 3 — FIGURE S3 Change in Quality of Life from before to during the pandemic per age group, in percentages. [file GBB-21-e12796-s003.png]
